# Assessing the Feasibility of the One-Minute Sit-to-Stand Test as a Substitute for the Six-Minute Walk Test in Type 2 Diabetes: A Cross-Sectional Study

**DOI:** 10.3390/jcm14124088

**Published:** 2025-06-10

**Authors:** Ahmad Alanazi

**Affiliations:** Department of Physical Therapy and Health Rehabilitation, College of Applied Medical Sciences, Majmaah University, Al Majmaah 11952, Saudi Arabia; aalanazi@mu.edu.sa

**Keywords:** one-minute sit-to-stand test, six-minute walk test, functional status, physical activity, diabetes mellitus

## Abstract

**Background/Objectives**: Type 2 Diabetes Mellitus (T2DM) is associated with reduced exercise capacity. The six-minute-walk test (6MWT) is the gold standard submaximal test but has practical challenges, such as space constraint, time dependency and feasibility in clinical settings. This study aimed to determine if the one-minute sit-to-stand (1 min STS) test could predict walking capacity, as assessed by the 6MWT, among individuals with T2DM. **Methods**: Ninety-five individuals with T2DM participated in this cross-sectional study. Participants performed 1 min STS and 6MWT tests and completed the Arabic version of the International Physical Activity Questionnaire (Ar-IPAQ). Pearson’s correlation was used to determine the correlation between one min STS and 6MWT tests. Multiple regression analysis was performed to determine whether 1 min STS predicted 6MWT while controlling for age, gender, BMI, IPAQ, and duration of diabetes. **Results**: Participants (mean age: 65.4 ± 6.4 years; 26.3% female; mean diabetes duration: 11.6 ± 6.4 years) demonstrated a significant moderate correlation between 1 min STS and 6MWT (r = 0.636, 95% CI [0.47–0.79], *p* < 0.001). Regression analysis revealed that the 1 min STS significantly predicted the 6MWT (β = 0.48, *p* < 0.001), regardless of age, gender, BMI, IPAQ, and duration of diabetes. **Conclusions**: The 1 min STS is inexpensive, simple to use, requires less space and materials that are widely available, and does not require experienced clinicians or researchers to administer. This test can be an alternative to the 6MWT for individuals with T2DM.

## 1. Introduction

Type 2 diabetes mellitus (T2DM) is a chronic metabolic disorder characterized by high blood glucose levels due to insulin resistance or inadequate insulin production [1]. It is the most common form of diabetes, accounting for more than 90% of all diabetes cases globally [2]. Approximately 10.5% of the world’s adult population (537 million people) will have diabetes by 2021, which is expected to increase to 783 million by 2045 [3]. Chronic hyperglycemia contributes to microvascular and macrovascular complications that can impair mobility, reduced muscle performance [4,5], and lead to earlier declines in physical function [6]. Chronic hyperglycemia induces mitochondrial dysfunction, advanced glycation end-products in skeletal muscle, and peripheral neuropathy, collectively impairing neuromuscular efficiency and oxygen utilization during exercise [7]. Regular evaluation of functional capacity can help identify patients at risk for mobility problems, enabling timely interventions to improve strength and endurance. Functional capacity assessment in individuals with T2DM is critical, given the high prevalence of physical deconditioning and increased risk of cardiovascular complications in this population. Functional capacity can be assessed using physical performance tests such as the six-minute walk test (6MWT) and the Timed Up and Go test.

One standard tool for assessing functional exercise capacity is the six-minute walk test (6MWT). The 6MWT is a submaximal exercise test used to evaluate aerobic endurance and mobility by measuring the distance an individual can walk on a flat surface within six minutes. The test has been utilized in various disorders, such as COPD, pulmonary arterial hypertension, and idiopathic pulmonary fibrosis, and in patients awaiting lung transplant [8] and T2DM [9]. Although the 6MWT is well established and reproducible, its limitations restrict its use, especially in routine clinical settings [10]. A major practical concern is the space requirement; standard guidelines recommend a 30 m hallway or similar open area to conduct the test properly [10]. Many clinics and offices lack such spaces, making it challenging to administer a true 6MWT outside specialized facilities. This spatial constraint not only affects the feasibility of the test but may also compromise its validity, as shorter tracks necessitate more turns, which can alter walking patterns and underestimate functional capacity [11]. This is particularly relevant in populations with diabetes, where functional testing is crucial for monitoring disease progression and physical performance [12]. Moreover, time constraints and patient fatigue in busy outpatient settings further limit its applicability. A functional test that requires less space and time and provides a meaningful measure of capacity would be highly useful. One such test is the one-minute sit-to-stand test (1 min STS), which has emerged as a promising alternative for measuring functional capacity in settings where the 6MWT is impractical. The 1 min STS is a test that requires a person to stand up from a chair and sit down as often as possible within one minute. The 1 min STS has several practical advantages, such as being easy to administer, time efficient, and requiring space for a chair. The 1 min STS assesses lower limb strength and endurance, which are critical for gait mechanics and energy expenditure during walking [13,14]. The performance on the 1 min STS may physiologically relate to free walking ability, as both require lower limb muscle strength, postural control, and cardiovascular endurance. STS performance reflects the functional integration of neuromuscular coordination and dynamic balance key components also involved in walking, especially over prolonged distances. A meta-analysis conducted in 2022 identified the STS test as the primary tool used to assess the effectiveness of exercise interventions in improving physical function among patients with sarcopenia and diabetes mellitus [15]. Peripheral neuropathy may disproportionately affect 6MWT performance due to balance challenges, whereas STS may better isolate muscular endurance [16].

This cross-sectional study was designed to assess the feasibility of the 1 min STS test as a substitute for the 6MWT in individuals with T2DM. The primary objective was to determine whether performance on the 1 min STS would predict functional exercise capacity as assessed by the 6MWT in a T2DM cohort. It was hypothesized that there will be a significant positive correlation between performance on the 1-minute Sit-to-Stand test and the 6-Minute Walk Test in individuals with type 2 diabetes, indicating that the STS test may serve as a valid proxy for assessing functional capacity in this population.

## 2. Materials and Methods

### 2.1. Design and Sample Size Calculation

This correlational cross-sectional study was conducted at the Qassim National Hospital and the Association of Al-Wafa Oasis for Elderly Support in Qassim City between May 2023 and May 2024 among individuals with T2DM. The study was approved before commencement by the Majmaah University Ethical Committee (Approval No. MUREC-May. 23/COM-2023/18-11). This study adhered to the principles of the Declaration of Helsinki, and all participants provided written informed consent.

Participants were recruited using a consecutive sampling method from the outpatient departments. Out of 176 eligible individuals approached, 95 agreed to participate, resulting in a participation rate of 53.9%. Efforts were made to minimize selection bias by recruiting from both clinical and community settings and applying consistent inclusion and exclusion criteria. However, selection bias may remain due to the voluntary nature of participation.

The sample size was calculated based on previous studies that reported a moderate correlations (r = 0.451) between 1 min STS and physical performance measures [17]. The power analysis (using G*Power) revealed that a minimum of 81 participants were needed with a power of 0.99 and an alpha level of 0.05.

### 2.2. Inclusion and Exclusion Criteria

Inclusion criteria were adults aged 18–75 years, with a confirmed diagnosis of T2DM (≥1-year duration), ambulatory without assistive devices, and cognitively capable of following test instructions. Exclusion criteria included acute cardiovascular or musculoskeletal conditions, neurological impairments, recent surgery within the past three months, or unstable diabetes (HbA1c > 10%).

### 2.3. Data Collection and Instruments

The participants’ demographic data, including age, gender, body mass index (BMI), and duration of diabetes diagnosis, were collected using a standardized data collection form. All research personnel underwent standardized training to administer the 6MWT and STS tests to enhance reliability. A pilot trial was conducted before data collection to ensure the instructions, measurement techniques, and equipment calibration consistency. The 6MWT was conducted according to the American Thoracic Society (ATS) guidelines [18]. The reliability and validity of the 6MWT have been reported in various disorders, such as measuring cardiovascular fitness among patients with T2DM [19] and as a tool for tele-assessment [20]. The participants wore normal shoes and were asked to walk back and forth along a 30 m corridor marked at 5 m intervals, and the total distance walked (m) was recorded. The participants were permitted to pause and take a break if necessary.

The 1 min STS tests were performed on a standard chair (height, 45 cm) with no arm supports, according to Bohannon’s guidelines [21]. Research has reported the reliability and validity of this test in patients with COPD [22], pulmonary hypertension [23] and artial fibrillation [24]. For the reliability and consistency of the test performance, each participant’s foot placement was standardized, and a safety protocol was in place to promptly address any adverse events. The participants were given demonstrations and allowed to perform practice trials to ensure familiarization with the tasks. For the 1 min STS test, participants were instructed to stand up and sit down on a chair as fast as possible for 1 min, and the number of repetitions was recorded. The number of sit-to-stand repetitions completed was also recorded. Both tests were performed in a randomized order, determined by computer-generated randomization, on the same days in morning with at least 15 min intervals to minimize fatigue-related bias [25]. All assessments were performed by trained research personnel blinded to the study hypotheses.

The Arabic short form of the International Physical Activity Questionnaire (IPAQ) [26] was used to assess physical activity; a seven-question survey designed to assess the occurrence and duration of different activities involved over the last 7 days. The IPAQ has been reported to be a reliable and valid tool to measure physical activity [27]. Participants were classified into low, moderate, or high physical activity levels and assessed as time multiplied by established metabolic equivalents (MET) for each activity based on the Ar-IPAQ scoring protocol.

### 2.4. Statistical Procedure

Statistical analyses were performed using IBM SPSS Statistics version 20. Descriptive statistics were calculated for demographic variables, including mean and standard deviation (SD) for continuous variables and frequency (n) and percentage (%) for categorical variables. Pearson’s correlation coefficient was used to examine the relationship between 6MWT performance and 1 min STS repetitions. To estimate the confidence interval for the Pearson correlation coefficient, the variables were first standardized (converted to z-scores), after which a linear regression analysis was performed. The resulting standardized beta coefficient, which corresponds to the Pearson correlation, was used to derive the confidence interval. Regression analysis was performed to predict 6MWT performance, incorporating predictors such as age, gender, BMI, 1-min-STS, years of diabetes mellitus. Assumption testing was conducted to verify multicollinearity (VIF < 10), normality of residuals (Shapiro–Wilk test), linearity (scatterplots of predictors against residuals), and homoscedasticity (scatterplots of standardized residuals against predicted values), ensuring the validity of the regression model.

## 3. Results

The sample consisted of 95 participants with a mean age of 65.38 ± 6.4 years. Of these, 70 participants (73.7%) were female. The mean body mass index (BMI) was 29.7 ± 4.7 kg/m^2^, and the mean duration of diabetes mellitus (DM) diagnosis was 11.55 ± 6.43 years. Functional capacity measured by the 1 min STS test showed a mean value of 11.58 ± 3.2 repetitions, and the mean distance covered in the 6-minute walk test (6MWT) was 342.91 ± 78.08 m. Regarding physical activity assessed using the IPAQ, 58 participants (61.05%) reported low levels, 27 (28.4%) moderate levels, and 10 (10.53%) high levels of physical activity (Table 1). Pearson correlation was conducted to examine the relationship between the 6MWT distance and the number of repetitions STS performed in 1 min. A significant positive correlation was observed between 1 min STS test and 6MWT, r = 0.636, *p* < 0.001.

Two models were evaluated in the regression analysis to predict the 6-minute walk test performance (AVG 6MWT). In Model 1, significant predictors included Age (β = −0.21, *p* = 0.015) and 1 min STS performance (β = 0.52, *p* < 0.001), indicating that younger age and better 1 min STS performance were associated with higher 6MWT distances. Gender and BMI did not significantly predict 6MWT in this model. Model 2 retained Age (β = −0.10, *p* = 0.292), 1 min STS (β = 0.48, *p* < 0.001), years of diabetes mellitus (DM) (β = −0.16, *p* = 0.066), and physical activity level (IPAQ) (β = 0.27, *p* = 0.001) as predictors. Age and gender showed non-significant trends in predicting the 6MWT. These findings suggest that physical performance in tasks like the 1 min STS and activity level are more robust predictors of 6MWT performance than age, gender, or BMI alone in this sample (Table 2).

## 4. Discussion

**Figure 1:**
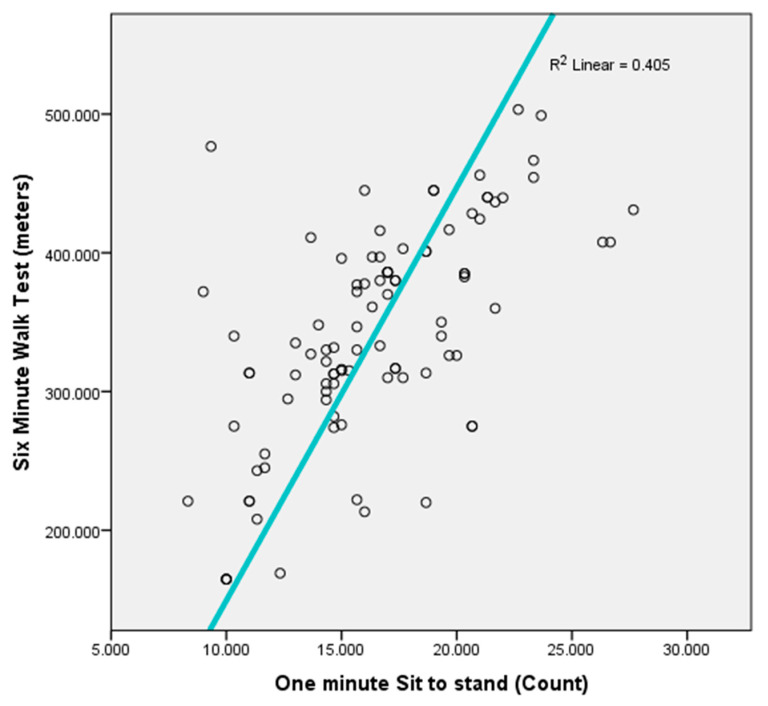
Scatter plot between one-minute STS and six-minute walk test.

This cross-sectional study assessed whether the 1 min STS test could serve as a feasible alternative to the 6MWT for estimating functional exercise capacity in individuals with T2DM. The data revealed that the 1 min STS test performance demonstrated a moderate positive correlation (r = 0.636, *p* < 0.001) with the 6MWT distance, underscoring its potential as a quick and accessible proxy measure of functional capacity (Figure 1). Moreover, regression analyses revealed that the 1 min STS significantly predicted 6MWT distance after controlling for demographic and clinical variables such as age, gender, body mass index (BMI), and duration of diabetes. Neuropathy may reduce 6MWT distance due to pain, while STS minimizes balance demands, offering a more accurate measure of strength. Given that the mean age of our sample was 65 years (range 40–70 years), the participants likely represented a relatively mix age group and less frail diabetic population. In older or more functionally impaired individuals, especially those over 70 years or with greater comorbid burden, the relationship between the 1STS and 6MWT may differ, as physical function is more severely affected.

The significant predictive ability of the 1 min STS test for 6MWT performance aligns with the growing body of literature suggesting that lower limb muscular endurance and power play a critical role in walking performance [28]. This strong association indicates that individuals who can complete more sit-to-stand repetitions in one minute are likely to walk farther over six minutes. Similarly, another study reported a positive correlation between the STS test and 6MWT in patients with cystic fibrosis [29]. Notably, while age and diabetes duration showed trends toward significance in regression modeling, their predictive impact was smaller than that of 1 min STS performance. This finding suggests that specific functional measures and overall physical activity engagement may weigh more heavily on daily mobility than chronological age.

Furthermore, the results expand on previous studies demonstrating that individuals with T2DM frequently exhibit compromised exercise tolerance due to peripheral neuropathy, reduced cardiovascular fitness, and a higher prevalence of comorbidities [30,31]. The 6MWT is a commonly used sub-maximal exercise test for measuring physical functional capacity; however, many clinical settings lack the space and time to administer it [10]. In contrast, the 1 min STS test is both space- and time-efficient, requiring only a standard chair. This study provides evidence that this simple test captures essential information regarding the functional capacity of patients with T2DM.

Several prior investigations in populations with cardiopulmonary disorders and healthy individuals have shown that 1 min STS correlates strongly with walking-based assessments, including the 6MWT [13,29,32]. For instance, Bohannon’s seminal work established the reliability and validity of STS tests in older adults and patients with chronic obstructive pulmonary disease [14,15]. Recent pulmonary hypertension and atrial fibrillation studies have also reported significant associations between STS performance and walking endurance [16,17].

An important strength of the 1 min STS is its minimal resource requirements. It is well-suited for primary care clinics, community health centers, and telehealth contexts where a 30 m corridor for the 6MWT may be unavailable. By offering a rapid snapshot of a patient’s lower extremity function, the 1 min STS test could be integrated into routine diabetic check-ups to monitor disease progression, guide exercise prescriptions, and identify patients requiring targeted rehabilitation. Incorporating this test into electronic medical records or diabetic care checklists could further standardize its use in clinical workflows. Lower limb strength correlated with health-related quality of life among community-dwelling elderly [33]. Moreover, incorporating STS assessments into diabetes management programs may facilitate early interventions to address functional decline. Exercise and rehabilitation strategies can then be personalized to improve lower body strength and endurance, ultimately enhancing walking capacity and overall quality of life.

### 4.1. Strengths and Limitations

A key strength of this study lies in using of standardized protocols for both the 6MWT and 1 min STS, administered by trained personnel blinded to the study hypotheses. This approach helps to minimize bias and enhances the internal validity of our findings. The sample size was calculated a priori based on the anticipated effect sizes and included a relatively diverse group of adults with T2DM.

However, several limitations should be noted. First, the cross-sectional design precludes any causal inferences regarding whether improving STS performance directly leads to better 6MWT outcomes. Second, although controlling for important covariates (age, BMI, IPAQ level, duration of diabetes) was considered, residual confounding from the unmeasured variables was possible. Third, this study was conducted at a single center, which may limit the generalizability of the findings. Although standardized recruitment procedures were used, the possibility of selection bias cannot be entirely ruled out, as individuals who agreed to participate may have differed in motivation or functional capacity from those who declined. Future studies involving multiple centers and varied population groups will help confirm and extend these results.

### 4.2. Future Directions

Prospective or longitudinal research examining changes in both STS and 6MWT over time could clarify whether improvements in the 1 min STS translate into gains in functional mobility. Intervention-based studies could investigate whether targeted exercise prescriptions to enhance STS performance also produce proportional increases in walking capacity. Additionally, further stratification of T2DM severity (e.g., glycemic control and the presence of diabetic complications) could refine the applicability of the test to specific subgroups. Future studies should explore the validity of the 1 min STS in more geriatric and frail cohorts. The functional capacity of individuals with diabetes is indeed often compromised by complications such as peripheral neuropathy, diabetic foot syndrome, and cardiovascular comorbidities. While this study did not stratify participants based on these specific complications, it is important to note that such conditions may influence the performance and sensitivity of both the 1 min STS and the 6MWT tests. Future research should investigate the comparative utility of these tests in these high-risk subgroups, where functional assessments may offer greater clinical insight and guide individualized rehabilitation strategies. Although participant preference was not formally assessed in this study, future research should explore perceived exertion, comfort, and acceptability of the 1 min STS versus the 6MWT tests. Understanding patient perspectives could inform test selection and implementation in primary care or resource-limited settings, where feasibility and patient compliance are key factors.

## 5. Conclusions

This study provides evidence that the 1 min STS test is a practical and valid tool for estimating functional capacity in adults with T2DM. Its strong correlation with the 6MWT suggests it captures the essential facets of lower extremity muscular endurance relevant to submaximal walking performance. Given its minimal resource requirements and ease of administration, the 1 min STS test can be a convenient alternative to the 6MWT in clinical and community settings, helping healthcare providers identify those at risk of functional decline and tailor interventions to improve overall metabolic and physical health.

## Figures and Tables

**Table 1:** Demographic data.

Variables	Total Sample (*n* = 95)	<10 Years (Duration of Diabetes) *n* = 53	>10 Years (Duration of Diabetes) *n* = 42	*p*-Value
Age, years, mean (SD)	65.38 (6.4)	55.9 (4.9)	61.5 (6.7)	0.001 *
Sex, Female, n (%)	70 (73.7)	40 (75.5)	30 (71.4)	0.001 **
BMI (kg/m^2^), mean (SD)	29.7 (4.7)	29.9 (5.4)	29.4 (3.7)	0.635 *
Duration of DM (yr), mean (SD)	11.55 (6.43)	6.7 (2.8)	17.6 (4.0)	0.001 *
1 min sit to stand test, mean (SD)	11.58 (3.2)	16.6 (4.1)	16.3 (4.1)	0.693 *
**6MWT, mean (SD)**	342.91 (78.08)	356.6 (77.4)	325.6 (76.2)	0.054 *
Ar-IPAQ n (%)	High 10 (10.53)	5 (9.4)	5 (11.9)	0.403 **
	Low 58 (61.05)	30 (56.6)	66.7 (66.7)	
	Moderate 27 (28.4)	18 (34.0)	9 (21.4)	

Abbreviations: ** Chi–square; * Independent sample *t*-test; DM = Diabetes mellitus; BMI = Body mass index; 6MWT = 6-minute walk test; Ar-IPAQ = Arabic version International Physical Activity Questionnaire Short form.

**Table 2:** Multiple linear regression analysis of the six-minute walk test as the dependent variable and demographic and the one-minute sit-to-stand test as predictors.

**Model 1**	**B**	**SE**	**Beta**	***p***	**95% CI**
Intercept	358.69	96.96	–	0.001	166.06, 551.32
Age (years)	–2.56	1.03	–0.21	0.015	–4.61, –0.52
Gender (female = 1)	–25.15	14.81	–0.14	0.093	–54.56, 4.27
BMI (kg/m^2^)	0.53	1.36	0.03	0.7	–2.17, 3.23
1 min STS (count)	9.82	1.76	0.52	0.001	6.33, 13.31
**Model 2**	**B**	**SE**	**Beta**	***p***	**95% CI**
Intercept	261.21	93.26	–	0.006	75.87, 446.55
Age (years)	–1.20	1.14	–0.10	0.292	–3.46, 1.05
Gender (female = 1)	–20.57	13.74	–0.12	0.138	–47.86, 6.73
BMI (kg/m^2^)	0.47	1.27	0.03	0.713	–2.06, 3.00
1 min STS (count)	9.11	1.65	0.48	0.001	5.83, 12.38
DM (years)	–1.97	1.06	–0.16	0.066	–4.08, 0.13
Ar-IPQ	31.04	8.59	0.27	0.001	13.96, 48.11

Abbreviations: B = unstandardized coefficient; SE = standard error; Beta = standardized coefficient; BMI = Body Mass Index; 1 min STS = 1-minute sit-to-stand test; DM (yr) = Duration of diabetes (years); Ar-IPQ = Arabic version International Physical Activity Questionnaire Short form.

## Data Availability

The data used in this study are available from the corresponding author on reasonable request.

## Abbreviations

The following abbreviations are used in this manuscript:

T2DM	Type 2 Diabetes Mellitus
6MWT	Six-Minute Walk Test
1 min STS	One-Minute Sit-to-Stand Test
IPAQ	International Physical Activity Questionnaire
Ar-IPAQ	Arabic Version of the International Physical Activity Questionnaire (Short Form)
BMI	Body Mass Index
DM	Diabetes Mellitus
COPD	Chronic Obstructive Pulmonary Disease
MET	Metabolic Equivalent
ATS	American Thoracic Society
HbA1c	Hemoglobin A1c
